# Influence of starch sources and dietary protein levels on intestinal functionality and intestinal mucosal amino acids catabolism in broiler chickens

**DOI:** 10.1186/s40104-019-0334-9

**Published:** 2019-04-05

**Authors:** Dafei Yin, Peter H. Selle, Amy F. Moss, Youli Wang, Xiaoyu Dong, Zhibin Xiao, Yuming Guo, Jianmin Yuan

**Affiliations:** 1 State key Laboratory of Animal Nutrition, College of Animal Science and Technology, China Agricultural University, Beijing, 100193 People’s Republic of China; 2 Poultry Research Foundation, The University of Sydney, Werombi Road, Camden, NSW 2570 Australia

**Keywords:** Amino acids, Broiler chickens, Enterocytes, Glucose, Starch

## Abstract

**Background:**

There is growing interest in carbohydrate and protein nutrition to enhance the efficiency of animal production. Reduced-crude protein diets depress environmental pollution and feeding cost, but the challenge to their adoption is maintaining digestive function and growth performance of birds. The present study was conducted to evaluate the influence of different dietary starch sources and protein levels on intestinal functionality and mucosal amino acid catabolism.

**Methods:**

Six dietary treatments, based on maize and soybean meal, were offered to 360 AA^+^ male chicks from 6 to 35 d post-hatch as a 3 × 2 factorial array. Either waxy rice or amylose was added to a conventional maize-soy diet to provide three sources of starch with different digestion rates and relatively high and low dietary protein levels. Growth performance, parameters of intestinal functionality and concentrations of free amino acid in the portal circulation were determined.

**Results:**

In the grower phase, starch source influenced (*P* < 0.02) weight gain as diets containing amylose supported significantly higher weight gains than waxy rice. Significant increase of ileal ATP concentrations and Na^+^/K^+^-ATPase activity were found in amylose treatment. Also, amylose decreased BrdU positive cell numbers and down-regulated mRNA expression for *CASP-3*. GOT activity in the ileum was higher (*P* < 0.01) in birds offered low protein diets and there was a trend (*P* = 0.057) for waxy rice as a starch source to increase ileal GOT activities. There was a significant influence on the concentration of seventeen amino acids in the portal circulation with tryptophan the one exception. Waxy rice as a starch source generated 13.6% and 22.4% numerically higher concentrations of non-essential amino acids than maize and amylose, respectively.

**Conclusions:**

Amino acid catabolism in the gut mucosa is subject to nutritional regulation. Given that amino acids can be spared from catabolism in the gut mucosa by supplementation of amylose, it follows their post-enteral availability would be improved and intestinal energy would be derived more efficiently from glucose.

## Background

Chicken-meat is becoming increasingly important in meeting the global demand for protein generated by an expanding human population; however, its sustainable production is confronted by rising feed ingredient costs. Reduced-crude protein diets have been shown to depress nitrogen excretion and have the potential to reduce feeding costs but the challenge to their adoption is maintaining digestive function and growth performance of birds [1]. Different carbohydrate (starch) sources in poultry diets provide an opportunity to formulate more efficient reduced-crude protein diets [2, 3].

Relative intestinal growth is driven by the amount of luminal nutrients including starch and protein that require digestion to permit absorption of glucose and amino acids. The energy demand of the small intestine is notably high which is partially due to the rapid renewal of the epithelium to maintain its function [4, 5]. Both amino acids, especially glutamate and glutamine, and glucose undergo catabolism in avian enterocytes to generate energy [6]. The post-enteral availability of digestible amino acids, or their concentrations in the portal circulation, is ultimately determined by their metabolic fate in the gut mucosa where amino acids may enter either anabolic or catabolic pathways within enterocytes. A large proportion of amino acids that are absorbed along the small intestine fail to enter the portal circulation and become available for protein accretion because of their extensive utilization in the gut mucosa via anabolic and catabolic pathways [7]. One potential mechanism to reduce amino acid catabolism by mucosal cells is to provide an alternative source of energy. There are indications in poultry that slowly digestible starch spares amino acids from catabolism [3] which is of particular importance for reduced-crude protein diets which axiomatically contain more starch than standard broiler diets.

Digestion rates of starch vary between feedstuffs; therefore, three different principal sources of starch (waxy rice, maize, amylose) were incorporated into the dietary treatments in this study. The potential glycaemic index (pGI) is indicative of the rate of starch digestion and glucose absorption. Giuberti et al. [8] reported that rice had a pGI of 80 and an amylose proportion in starch of 23.3%; whereas, maize had a lower pGI of 39.5 with 31.1% amylose. Lowering protein concentrations will reduce feed costs; therefore, diets were formulated to standard and relatively low protein levels. This approach was adopted because there is intense interest in developing reduced-crude protein diets, which if successful, will lead to more sustainable chicken-meat production.

The post-enteral availability of amino acids is an absolute pre-requisite for protein synthesis and growth of broiler chickens. The pivotal question posed by Reeds et al. [9] was whether or not amino acid catabolism in the gut mucosa is subject to nutritional regulation. It may be possible to manipulate the ‘catabolic ratio’ of amino acids to glucose in the gut mucosa in poultry via dietary strategies to increase the post-enteral availability of amino acids. Therefore, the purpose of this study was to investigate mechanisms underlying the influence of dietary starch sources on post-enteral amino acid availability. Our hypothesis is that different dietary starch sources and protein levels have the capacity to influence intestinal functionality and reduce amino acid catabolism, which would be reflected in concentrations of free amino acids in the portal circulation.

## Methods

### Experimental design

The experimental design comprised a 3 × 2 factorial array of dietary treatments with starter (6 to 20 d post-hatch) and grower (21 to 35 d post-hatch) diets containing three sources of starch and two protein levels. Essentially, the different starch sources consisted of waxy rice, maize grain and amylose and protein levels were adjusted by maize gluten meal inclusions. The composition of the six starter and six grower diets are shown in Table 1.

**Table 1:** Feed ingredients and nutrient composition of experiment diets

Feed ingredients	Starter (6 to 20 d post-hatch)						Finisher (21 to 35 d post-hatch)					
Maize	325	330	600	600	520	530	352	353	645	642	572	573
Waxy rice	270	270	0	0	0	0	295	295	0	0	0	0
Amylose (97%)	0	0	0	0	70	70	0	0	0	0	75	75
Corn gluten meal	64.6	0	32	0	46.8	0	75.5	0	38.4	0	71.9	0
Soybean meal	265	296	296	281	289	294	207	256	247	241	210	254
Soy oil	24	37	18	35	25.2	38.1	28.3	43	24.6	42	29.3	44
Dicalcium phosphate	15.9	16	13.9	14.4	14.4	14.7	15.4	15.4	13.3	13.7	13.9	14
Limestone	12.6	12.4	13.6	13.5	13.4	13.2	11.6	11.2	12.4	12.3	12.4	12.1
Sodium chloride	3.5	3.5	3.5	3.5	3.5	3.5	3.5	3.5	3.5	3.5	3.5	3.5
Lysine HCl	3.3	2.9	2.5	3.3	2.7	3.1	4	2.9	2.8	3.5	4.1	3.2
*DL*-methionine	1.6	2.8	1.6	2.7	1.6	2.8	1.3	2.8	1.4	2.5	1.2	2.6
*L*-threonine	0.6	1.4	0.7	1.7	0.7	1.6	0.4	1.1	0.5	1.4	0.7	1.3
*L*-serine	0	0	0	0.1	0	0.1	0.2	0.2	0.2	0.3	0.3	0.3
*L*-arginine	0.2	0.3	0	1.1	0.3	0.9	0.3	0.1	0	0.9	0.9	0.8
Choline chloride (50%)	2.5	2.5	2.5	2.5	2.5	2.5	2	2	2	2	2	2
Vitamin mineral premix^a^	2.3	2.3	2.3	2.3	2.3	2.3	2.2	2.2	2.2	2.2	2.2	2.2
Antioxidant	0.2	0.2	0.2	0.2	0.2	0.2	0.2	0.2	0.2	0.2	0.2	0.2
Zinc bacitracin (10%)	0.4	0.4	0.4	0.4	0.4	0.4	0	0	0	0	0	0
Colistin sulphite (10%)	0.1	0.1	0.1	0.1	0.1	0.1	0	0	0	0	0	0
Flavomycin	0	0	0	0	0	0	0.2	0.2	0.2	0.2	0.2	0.2
Phytase	0.2	0.2	0.2	0.2	0.2	0.2	0.2	0.2	0.2	0.2	0.2	0.2
Zeolite	3.4	16.6	7.2	32.9	1.3	17.4	0.7	11	6.1	32.1	0	11.4
Titanium oxide	5	5	5	5	5	5	0	0	0	0	0	0
Calculated analysed												
Metabolisable energy, MJ/kg	12.37	12.33	12.33	12.37	12.33	12.33	12.75	12.75	12.75	12.75	12.75	12.75
Crude protein	210	185	210	185	210	185	195	170	195	170	195	170
Calcium	10.9	10.9	11.1	11.1	11.0	11.0	10.2	10.2	10.3	10.4	10.3	10.4
Total phosphorus	5.57	5.51	5.57	5.42	5.53	5.42	5.25	5.23	5.28	5.13	5.16	5.14
Available phosphorus	4.91	4.96	4.70	4.70	4.73	4.74	4.74	4.79	4.52	4.52	4.51	4.57
Lysine	11.3	11.3	11.3	11.3	11.2	11.2	10.5	10.4	10.4	10.5	10.6	10.4
Methionine	4.7	5.2	4.5	5.1	4.6	5.2	4.3	5.1	4.2	4.7	4.1	4.8
Methionine + Cysteine	7.5	7.7	7.3	7.5	7.3	7.6	6.9	7.3	6.8	7.0	7.0	7.1
Threonine	7.2	7.4	7.3	7.5	7.3	7.5	6.3	6.6	6.5	6.7	6.5	6.7
Analysed starch	385	358	361	370	382	351	419	387	388	396	384	379

^a^Provided per kg diet: vitamin A (as retinyl acetate), 12,500 IU; cholecalciferol, 2,500 IU; vitamin E (as *DL*-α-tocopherol acetate), 18.75 mg; menadione, 2.65 mg; thiamine, 2.5 mg; riboflavin, 6.0 mg; pyridoxine, 4.9 mg; pantothenic acid, 12 mg; niacin, 50 mg; folic acid, 1.25 mg; biotin, 0.0325 mg; cobalamine, 0.025 mg Cu (CuSO_4_·5H_2_O), 8 mg; Zn (ZnSO_4_·H_2_O), 75 mg; Fe (FeSO_4_·H_2_O), 80 mg; Mn (MnSO_4_·H_2_O), 100 mg; Se (Na_2_SeO_3_), 0.30 mg; I (Ca(IO_3_)·H_2_O), 0.35 mg

### Diet preparation

Before the diets were prepared essential amino acid levels of key feedstuffs were analyzed so that the formulated experimental diets met NY/T33–2004. The nutrient specifications of the experimental diets are shown in Table 2. The starter diets were extruded at 70 °C with a twin-screw extruder with a screw speed of 400 r/min and water addition rates of 15 kg/h. The grower diets were steam-pelleted at a temperature of 65 °C with a conditioner residence time of 20 s.

**Table 2:** Primers used for quantitative real-time PCR

Gene name	Accession number	Forward sequence (5′ to 3′)	Reverse sequence (5′ to 3′)
*β-actin*	NM_205518	GAGAAATTGTGCGTGACATCA	CCTGAACCTCTCATTGCCA
*mTOR*	XM_417614.5	GAAGTCCTGCGCGAGCATAAG	TTTGTGTCCATCAGCCTCCAGT
*GLUT2*	Z22932	CCGCAGAAGGTGATAGAAGC	ATTGTCCCTGGAGGTGTT
*SGLT1*	XM_415247	AGATTTGGAGGGCAGAGGAT	GCCCAAAGAGATTTGGATGA
*PepT1*	AY029615	TACGCATACTGTCACCATCA	TCCTGAGAACGGACTGTAAT
*APN*	NM_204861	GATCCACAGCAACAAGCTGA	TACTGCGTGTTGGTCTCCAG
*rBAT*	XM_426125	AAGGAGCTGAAGGGCTTACC	ATTTCATCTTGGCTGCTGGT
*ATB* ^0,+^	XM_426267	CCTGCTCATCTTGTTGGTGA	GCATCTTTCCAAACCTCTGC
*B* ^0^ *AT1*	XM_419056	TATCCTGGCTGGGTCTATGC	AGGCCTGTACGATCCCTTCT
*EAAT3*	XM_424930	TGATTGTTCTGAGCGCTGTC	TACCAAAGGCATCTCCCAAG
*CAT1*	XM_417116	CACATGGATACGGTTTGCAG	GTCCATGCTTCTCTCCGTGT
*y* ^*+*^ *LAT1*	XM_418326	CACCAGTCCCTGCTCTTCTC	CTGCAATAGACAAGCCCACA
*LAT1*	CD217821	TACCTGCTGAAGCCCATCTT	ACGGGTAGCAGCTTTCACAC
*CASP-3*	NM_204725.1	ACACGCCAGGAAACTTGAAC	ACACGCCAGGAAACTTGAAC

### Bird management

A total of 360 Arbor Acres plus broilers were obtained from a commercial hatchery and at 6 d post-hatch were randomly assigned to 6 treatments, each consisting of 6 replicates with 10 chicks per replicate cage. The overall feeding period was from 6 to 35 d post-hatch where the chickens had unrestricted access to feed and water under a 23-h “lights-on” regime. Feed consumption and body weight for each replicate cage of birds were monitored at 6, 20 and 35 d post-hatch. Weight gains, feed intakes and feed conversion ratios (FCR) were determined for the nominated feeding intervals.

### Sample collection and chemical analyses

At day 35, one bird per replicate cage, or six birds per treatment, was selected and euthanized by an intravenous injection of 5% sodium pentobarbitone. Immediately following euthanasia, the abdominal cavity was opened and blood samples withdrawn from the anterior mesenteric vein. Plasma samples were stored at − 40 °C after centrifugation for quantification of amino acids. For amino acid analysis, 50-μL frozen plasma samples were thawed at 4 °C and mixed with equal volume of deproteinized using 7.5% (*w*/*v*) trichloroacetic acid, then centrifuged at 10,000×*g* for 15 min at 4 °C. The amino acid concentration of deproteinized plasma was determined by ion exchange chromatography with an L8800 high-speed AA analyzer (Hitachi, Tokyo) [10].

### Mucosal enzyme activities and ATP content

Also, 10 cm jejunal and ileal segments were opened and flushed, and mucosal samples were collected, then rapidly frozen in liquid nitrogen and stored at − 80 °C for analysis of enzyme activities and ATP. The activity level of glutamic-oxaloacetic transaminase (EC 2.6.1.1) was determined using the assay kit from Nanjing Jiancheng Bioengineering Institute (Art. No. C009–2, C010–2, Nanjing, China). The Karmen’s unit, defined as a decreased of 0.001 in absorbance min^-1^ at 340 nm and 250 nm, was used to report glutamic-oxaloacetic transaminase activity. The piece of mucosa excised for the determination of ATP was immediately placed in physiological saline solution, washed, blotted with filter paper, and weighed and then homogenized in iced trichloroacetic acid solution. The ATP content of the intestinal mucosa samples was determined by assay kits from Nanjing Jiancheng Bioengineering Institute (Art. No. A095). The results were expressed in milligrams percent of ATP. The activity levels of Na^+^/K^+^-ATPase and citrate synthase (EC 2.3.3.1) (CS) were analyzed with commercially available kits (Art. No. A070–2, A108, Beijing Solarbio Science & Technology Co., Ltd).

### Immunohistochemistry

The 2 cm intestinal segments were flushed and then fixed in 4% paraformaldehyde solution for analysis of intestinal morphology and cell apoptosis and proliferation. After 24 h of fixation paraffin sections were pretreated with 0.03% pronase in 0.05 mol/L Tris-HCl buffer (pH 7.6) for 3 min, rinsed in PBS, treated with 1.5% normal horse serum in PBS for 20 min, incubated with mouse anti-BrdU antibody for 14 h at 4 °C, washed in PBS, incubated with biotinylated secondary antibody for 40 min, and treated with ABC-peroxidase kit (Vector Laboratories) for 60 min. Finally, BrdU-labeled cells were visualized with 3,3′-diaminobenzidine tetrahydrochloride (DAB) and counterstained with Mayer’s hematoxylin or methyl green. Apoptotic cells were detected by the terminal deoxynucleotidyl-transferase (TdT)-mediated deoxyuridine triphosphate biotin (dUTP) nick-end labeling (TUNEL) method by using the DeadEnd Colorimetric TUNEL System.

### RNA extraction and relative qPCR for broiler transporters

Two 0.5 cm sections of small intestine were sampled and frozen in liquid nitrogen for mRNA determination. Total RNA isolation was carried out using TRIzol reagent (Invitrogen Life Technologies, Carlsbad, CA) according to the manufacturer’s instructions. The concentration and purity of total RNA were monitored by measuring its optical density at 260 and 280 nm. One microgram of total RNA was reverse transcribed with a reverse transcription kit (Invitrogen Life Technologies) according to the manufacturer’s instructions. A quantitative real-time PCR assay was performed with the 7500 fluorescence detection system (Applied Biosystems, Foster City, CA) according to optimized PCR protocols using the SYBR-Green PCR kit (Takara Biomedical Technology Co., Ltd.). Nested primers were designed (Table 2) within cloned chicken cDNA sequences with the Primer Express software, optimized for use with Applied Biosystems Real-Time PCR Systems. The PCR conditions were an initial denaturation step at 95 °C for 10 min, 40 cycles at 95 °C for 30 s and annealing and extension temperature at 60 °C for 1 min, and a final extension step of 72 °C for 10 min. To confirm amplification specificity, we subjected the PCR products from each primer pair to a melting curve analysis and subsequent agarose gel electrophoresis. Gene expression was quantified using the comparative threshold cycle method [11], and the data were expressed as the value relative to the high protein and maize starch treatment group.

### Statistical analysis

Experimental data derived from six replicate cages per treatment was analysed using the IBM® SPSS® Statistics 24 program (IBM Corporation. Somers, NY). The experimental unit was each replicate cage and statistical procedures included univariate analyses of variance using the general linear models procedure, Pearson correlations and linear and quadratic regressions. A probability level of less than 5% by a two-tailed test was considered to be statistically significant.

## Results

### Growth performance

The effects of dietary treatments on growth performance are shown in Table 3. Overall, from 6 to 35 d post-hatch, high protein diets supported greater weight gains (*P* < 0.025). Starch source influenced FCR (*P* < 0.005) where both waxy rice (1.550) and amylose (1.545) were superior to maize (1.597). In the starter phase, starch source influenced weight gain (*P* < 0.005) where waxy rice was superior to amylose and maize. Birds offered low-protein diets had higher feed intakes (*P* < 0.025). Starch source significantly influenced FCR (*P* < 0.001) where waxy rice was statistically superior to amylose which was, in turn, superior to maize. Birds offered high-protein diets displayed improvements in FCR (*P* < 0.001) in comparison to low-protein diets. In the grower phase, starch source influenced (*P* < 0.02) weight gain as diets containing amylose supported significantly higher weight gains than waxy rice. Also, high protein diets increased feed intakes (*P* < 0.05) in comparison to low protein diets.

**Table 3:** Effects of dietary treatments on growth performance in the starter phase (6 to 20 d post-hatch), grower phase (21 to 35 d post-hatch) and overall (from 6 to 35 d post-hatch)^d^

Treatment		Starter phase			Grower phase			Overall		
Starch	Protein	Gain, g/bird	Intake, g/bird	FCR	Gain, g/bird	Intake, g/bird	FCR	Gain, g/bird	Intake, g/bird	FCR
Waxy rice	High	720	971	1.349	1488	2539	1.655	2222	3546	1.555
	Low	734	1035	1.411	1462	2356	1.613	2169	3392	1.545
Maize	High	703	1099	1.446	1537	2527	1.636	2231	3529	1.579
	Low	686	1044	1.522	1511	2482	1.644	2196	3547	1.615
Amylose	High	714	1018	1.426	1576	2502	1.588	2289	3520	1.527
	Low	691	1019	1.476	1532	2459	1.620	2208	3478	1.564
SEM		4.49	7.46	0.0113	11.62	21.91	0.0071	16.16	26.84	0.0073
Main effects: Starch										
Waxy rice		727^a^	1003	1.380^a^	1475^b^	2448	1.634	2195	3649	1.550^a^
Maize		694^b^	1027	1.484^c^	1524^ab^	2505	1.640	2214	3538	1.597^b^
Amylose		702^b^	1018	1.451^b^	1554^a^	2481	1.604	2249	3499	1.545^a^
Protein										
High		712	999^b^	1.410^b^	1533	2523^a^	1.626	2247^a^	3532	1.553
Low		704	1033^a^	1.470^a^	1502	2433^b^	1.626	2191^b^	3472	1.557
Significance (*P*-value)										
Starch		0.004	0.370	< 0.001	0.017	0.551	0.072	0.182	0.610	0.002
Protein		0.279	0.020	< 0.001	0.141	0.039	0.966	0.024	0.290	0.074
Starch × Protein		0.117	0.182	0.747	0.924	0.302	0.076	0.713	0.442	0.179

^d^Values are the mean and pooled standard error of the mean (SEM), *n* = 6 chicken/group

^a-c^means within each group with different letter designations differ (*P* < 0.05)

### mRNA expression for glucose transporters

**Fig. 1:**
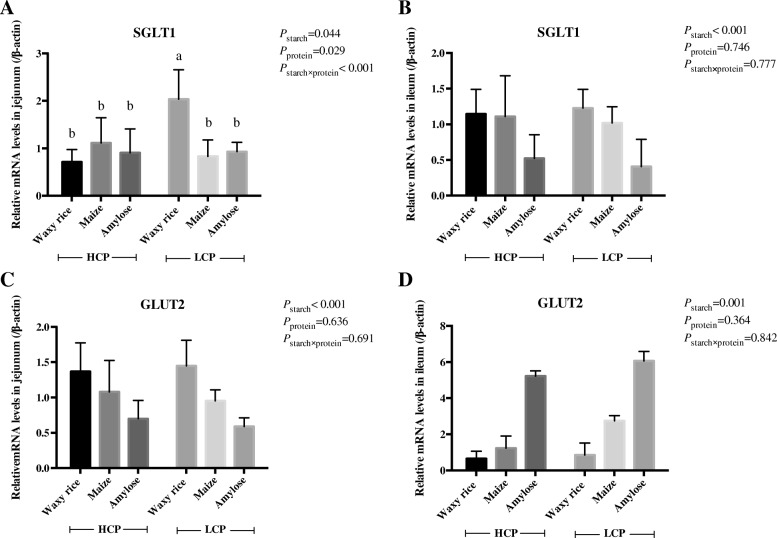
The effects of dietary starch source and high and low protein levels on relative mRNA expression for *SGLT-1* (**a** and **b**) and *GLUT-2* (**c** and **d**) in the jejunum and ileum at 35 d post-hatch. The data shown were means ± SEM, *n* = 6. ^a-b^ means within each group with different letter designations differ (*P* < 0.05). HCP means high protein level, LCP means low protein level

The effects of dietary treatments on relative mRNA expression for the two glucose transporters along the small intestine at 35 d post-hatch are shown in Fig. 1. There was an interaction (*P* < 0.001) for *SGLT-1* in the jejunum where birds offered low protein, waxy rice diets had the highest level of mRNA expression across the six treatments. There was no interaction in the ileum but starch source influenced (*P* < 0.001) expression where both waxy rice and maize starch sources generated higher levels than amylose. Starch source influenced (*P* < 0.001) mRNA expression for *GLUT-2* in both the jejunum and ileum. In the jejunum expression from waxy rice exceeded maize and amylose irrespective of dietary protein level but in the ileum the pattern of responses was an exact opposite where amylose as the starch source clearly generated higher mRNA expressions.

### mRNA expressions for PepT-1 and amino acid transporters

**Fig. 2:**
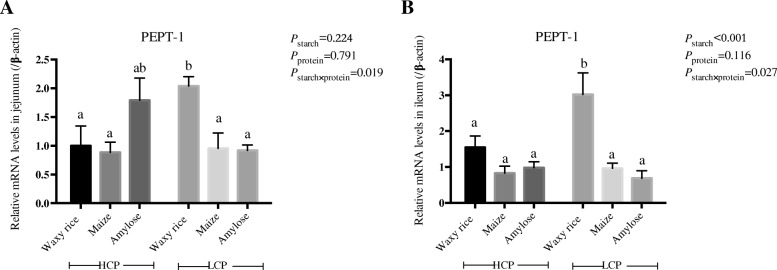
The effects of dietary starch source and high and low protein levels on relative mRNA expression for *PepT-1* in the jejunum (**a**) and ileum (**b**) at 35 d post-hatch. The data shown were means ± SEM, *n* = 6. ^a-b^ means within each group with different letter designations differ (*P* < 0.05). HCP means high protein level, LCP means low protein level

The influence of dietary treatments on relative mRNA expression for *PepT-1* in the jejunum and ileum is shown in Fig. 2 where significant interactions (*P* < 0.05) were observed in jejunum (*P* < 0.05) and ileum. In both instances, these interactions stemmed from substantial increases in mRNA expression with the transition from high to low protein levels in diets containing waxy rice as a starch source.

The influence of dietary treatments on relative mRNA expression for eight amino acid transporters in the ileum is shown in Table 4. Starch source in diets significantly influenced mRNA expressions for seven amino acid transporters. Expression of *ATB*^0,+^ was significantly lower in amylose diets than in waxy rice and maize diets which was also the case for *APN* and *rBAT*. *EAAT3* expression was significantly lower in amylose diets than in waxy rice diets. *CAT1* expression was significantly greater in maize diets than in amylose diets. *B*^0^*AT1* expression was greater in waxy rice diets than in maize diets and, in turn, amylose diets. Expression of *y*^*+*^*LAT1* was greater in waxy rice diets than in both maize and amylose diets. In contrast, mRNA expressions for *EAAT3* and *LAT1*, were significantly increased by low protein diets.

**Table 4:** Influence of dietary treatments on relative mRNA expression of amino acid transporters in ileum of broiler chickens at 35 d post-hatch^c^

Treatment		Amino acid transporters							
Starch	Protein	*ATB* ^0,+^	*EAAT3*	*CAT1*	*APN*	*rBAT*	*B* ^0^ *AT1*	*y* ^*+*^ *LAT1*	*LAT1*
Waxy rice	High	1.41	1.35	1.14	1.04	0.89	1.57	1.93	0.81
	Low	0.94	1.63	0.78	1.04	0.7	2.06	1.85	1.17
Maize	High	1.18	0.86	1.07	1.15	1.02	1.06	1.02	1.07
	Low	1.10	1.35	1.24	1.08	0.79	1.08	0.95	1.14
Amylose	High	0.47	0.77	0.62	0.59	0.49	0.59	0.90	0.85
	Low	0.48	0.92	0.77	0.43	0.42	0.57	1.02	1.74
SEM		0.124	0.085	0.072	0.085	0.051	0.110	0.104	0.097
Main effects: Starch									
Waxy rice		1.18^b^	1.49^b^	0.96^ab^	1.04^b^	0.80^b^	1.82^c^	1.89^b^	0.99
Maize		1.14^b^	1.11^ab^	1.16^b^	1.11^b^	0.91^b^	1.07^b^	0.99^a^	1.10
Amylose		0.48^a^	0.85^a^	0.70^a^	0.51^a^	0.45^a^	0.58^a^	0.96^a^	1.30
Protein									
High		1.02	0.99^b^	0.94	0.92	0.80	1.07	1.28	0.91^b^
Low		0.84	1.30^a^	0.93	0.85	0.64	1.24	1.27	1.35^a^
Significance (*P*-value)									
Starch		0.036	0.003	0.029	0.006	< 0.001	< 0.001	< 0.001	0.373
Protein		0.446	0.040	0.921	0.636	0.050	0.270	0.958	0.018
Starch × Protein		0.669	0.631	0.202	0.910	0.665	0.313	0.845	0.170

^c^Values are the mean and pooled standard error of the mean (SEM), *n* = 6 chicken/group

^a-b^means within each group with different letter designations differ (*P* < 0.05)

### ATP, Na^+^/K^+^-ATPase and citrate synthase activities

**Fig. 3:**
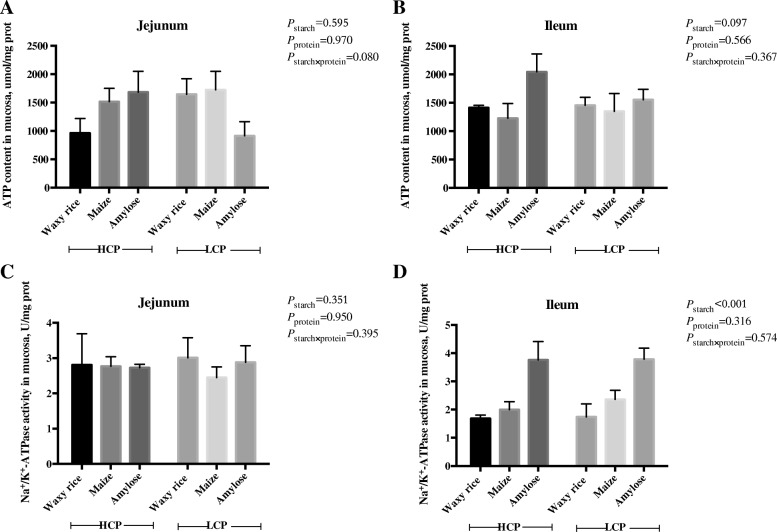
The effects of dietary starch source and high and low protein levels on ATP activity (**a** and **b**) and Na^+^/K^+^-ATPase activity (**c** and **d**) in the jejunal and ileal mucosa at 35 d post-hatch. The data shown were means ± SD, *n* = 6. HCP means high protein level, LCP means low protein level

The effects of dietary starch source and protein levels on ATP activity and Na^+^/K^+^-ATPase activity in the jejunal and ileal mucosa at 35 d post-hatch are shown in Fig. 3. In the ileum, starch source had a highly significant (*P* < 0.001) influence on Na^+^/K^+^-ATPase activity. Sodium pump activity was greatest in birds offered diets containing amylose, intermediate with maize, and least with birds offered waxy rice as the starch source. Dietary treatments did not significantly influence sodium pump activity in the jejunum or ATP activity in either small intestinal segment.

**Fig. 4:**
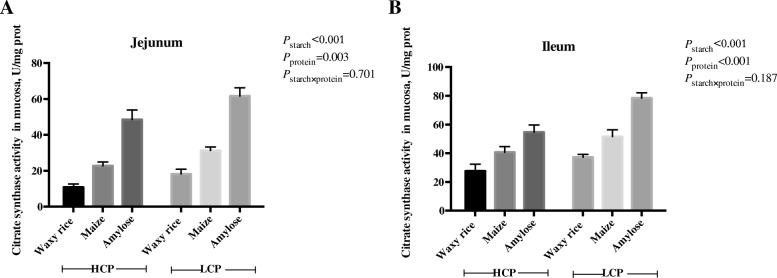
The effects of dietary starch source and high and low protein levels on citrate synthase activity in the jejunal (**a**) and ileal (**b**) mucosa at 35 d post-hatch. The data shown were means ± SD, *n* = 6. HCP means high protein level, LCP means low protein level

Treatment effects on citrate synthase activity are shown in Fig. 4. Both starch source (*P* < 0.001) and protein level (*P* < 0.005) similarly influenced citrate synthase activity in the jejunum and ileum. Amylose diets generated the most, maize was intermediate, and waxy rice diets generated the least citrate synthase activity in both small intestinal segments.

### Proliferation, apoptosis and mRNA expression for *CASP-3* and *mTOR*

**Fig. 5:**
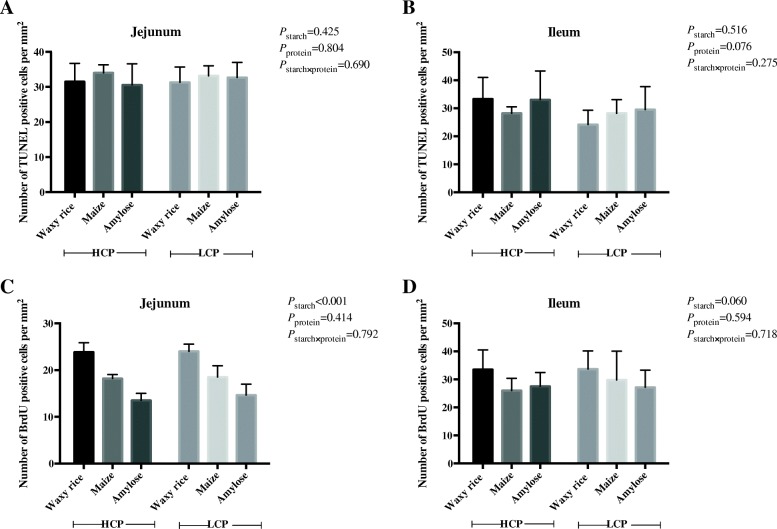
The effects of dietary starch source and high and low protein levels on the number of TUNEL positive cells (**a** and **b**) and BrdU positive cells (**c** and **d**) in the jejunum and ileum at 35 d post-hatch. The data shown were means ± SD, *n* = 6. HCP means high protein level, LCP means low protein level

The effects of dietary treatments on TUNEL positive cells and BrdU positive cells in the jejunum and ileum mucosa are shown in Fig. 5. Starch sources influenced (*P* < 0.001) BrdU positive cell numbers in the jejunum where diets containing waxy rice generated the highest numbers. Significant differences in BrdU positive cell numbers were not observed in the ileum but the response pattern was similar and the effect of waxy rice as a starch source approached significance. The numbers of TUNEL positive cells were not statistically influenced by treatment in either the jejunum or ileum.

**Fig. 6:**
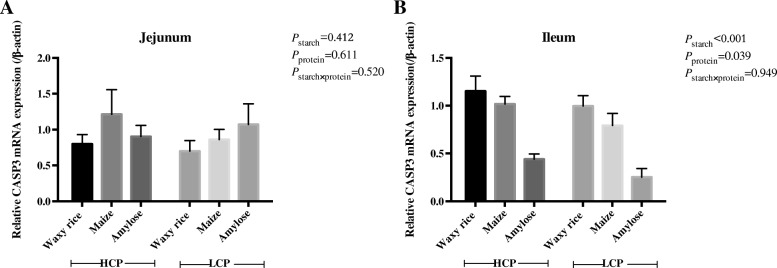
The effects of dietary starch source and high and low protein levels on relative mRNA expression for *CASP-3* in the jejunum (**a**) and ileum (**b**) at 35 d post-hatch. The data shown were means ± SEM, *n* = 6. HCP means high protein level, LCP means low protein level

The effects of dietary starch source and high and low protein levels on mRNA expression of *CASP-3* are shown in Fig. 6. There were no significant treatment effects in the jejunum. However, in the ileum diets containing waxy rice generated the highest mRNA expression. *CASP-3* mRNA expression was significantly greater in high protein diets without any indication of an interaction.

**Fig. 7:**
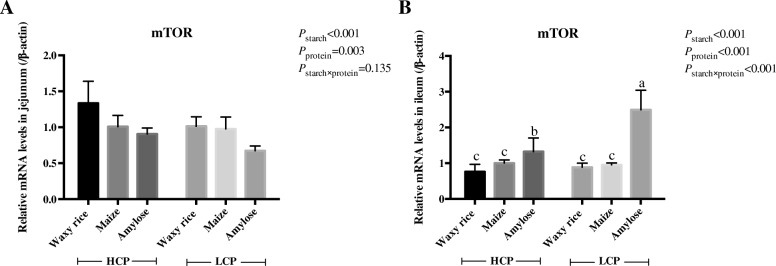
The effects of dietary starch source and high and low protein levels on mRNA expression for *mTOR* in the jejunum (**a**) and ileum (**b**) at 35 d post-hatch. The data shown were means ± SEM, *n* = 6. ^a-b^ means within each group with different letter designations differ (*P* < 0.05). HCP means high protein level, LCP means low protein level

The effects of dietary treatments on relative mRNA expression for *mTOR* in the jejunum and ileum are shown in Fig. 7. Both starch sources (*P* < 0.001) and protein levels (*P* < 0.005) influenced mRNA expression in the jejunum. The combination of waxy rice starch and high protein diets generated the highest levels while the combination of amylose and low protein diets generated the least mRNA expression for *mTOR*. There was a treatment interaction (*P* < 0.001) for *mTOR* expression in the ileum. Diets containing amylose as a starch source generated the greatest *mTOR* expression in low protein diets followed by amylose in high protein diets. Amylose diets supported significantly greater *mTOR* expression than diets containing either maize of waxy rice as the main starch source.

### Intestinal GOT activities and free amino acid concentrations in portal circulation

**Fig. 8:**
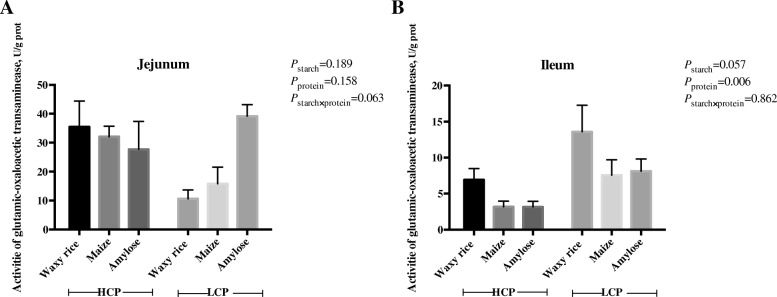
The effects of dietary starch source and high and low protein levels on glutamic-oxaloacetic transaminase activity in the jejunal (**a**) and ileal (**b**) mucosa at 35 d post-hatch. The data shown were means ± SD, *n* = 6. HCP means high protein level, LCP means low protein level

The effects of dietary treatments on the activity of glutamic-oxaloacetic transaminase in the jejunum and ileum are shown in Fig. 8. GOT activity in the ileum was higher (*P* < 0.01) in birds offered low protein diets and there was a trend (*P* = 0.057) for waxy rice as a starch source to increase ileal GOT activities which closely approached significance. Significant treatment effects were not observed in the jejunum.

Free concentrations of ten essential amino acids in plasma taken from the anterior mesenteric vein are shown in Table 5 where there were significant treatment effects for nine amino acids but not tryptophan. Significant treatment interactions were observed for six amino acids; whereas, protein levels significantly influenced concentrations of arginine, phenylalanine and threonine as a main effect. Arginine and threonine concentrations were increased while phenylalanine concentrations were decreased in low protein diets (*P* < 0.05).

**Table 5:** Effects of dietary treatments on concentrations (nmol/L) of free essential amino acids in plasma from the portal circulation (anterior mesenteric vein) at 35 d post-hatch^d^

Treatment		Arginine	Histidine	Isoleucine	Leucine	Lysine	Methionine	Phenyl- alanine	Threonine	Tryptophan	Valine
Starch	Protein										
Waxy rice	High	394	71^a^	114^ab^	327^cd^	234^a^	90^ab^	190	493	73	205^bc^
	Low	514	60^a^	121^b^	186^a^	264^ab^	113^b^	149	766	79	167^ab^
Maize	High	388	100^b^	126^b^	349^d^	227^a^	92^ab^	200	553	70	233^c^
	Low	365	58^a^	80^a^	220^ab^	194^a^	96^ab^	139	761	71	132^a^
Amylose	High	336	64^a^	112^ab^	295^cd^	218^a^	74^b^	187	578	72	174^ab^
	Low	455	71^a^	118^b^	270^bc^	347^b^	151^c^	180	951	83	181^b^
SEM		16.96	3.87	4.93	12.68	13.85	6.04	6.54	49.53	2.06	8.15
Main effects: Starch											
Waxy rice		454	66	117	256	249	102	170	630	76	186
Maize		377	79	103	285	210	94	169	657	70	183
Amylose		396	68	115	282	282	112	183	765	78	178
Protein											
High		373^a^	78	117	324	226	85	192^b^	541^a^	72	204
Low		445^b^	63	106	225	268	120	156^a^	826^b^	77	160
Significance (*P*-value)											
Starch		0.099	0.162	0.379	0.298	0.064	0.264	0.520	0.452	0.335	0.853
Protein		0.020	0.018	0.244	< 0.001	0.095	0.001	0.004	0.004	0.179	0.002
Starch × Protein		0.082	0.008	0.031	0.010	0.034	0.008	0.155	0.752	0.585	0.004

^d^Values are the mean and pooled standard error of the mean (SEM), *n* = 6 chicken/group

^a-c^means within each group with different letter designations differ (*P* < 0.05)

Dietary treatments effects on concentrations of eight free non-essential amino acids and total non-essentialamino acids in the portal circulation are shown in Table 6. Starch source significantly influenced glutamine (*P* < 0.05) concentrations, protein levels significantly influenced alanine, asparagine, and glutamic acid as main effects. Treatment interactions were observed for concentrations of aspartic acid (*P* < 0.05), glycine (*P* < 0.005), serine (*P* < 0.025) and tyrosine (*P* < 0.05).

**Table 6:** Effects of dietary treatments on concentrations (nmol/L) of free non-essential amino acids and total free amino acids in plasma from the portal circulation (anterior mesenteric vein) at 35 d post-hatch^d^

Treatment		Alanine	Aspartic acid	Asparagine	Glutamic acid	Glutamine	Glycine	Serine	Tyrosine	Total
Starch	Protein									
Waxy rice	High	882	190^b^	30	379	961	878^bc^	589^a^	263^b^	4173
	Low	1163	158^ab^	60	285	1098	869^bc^	988^b^	186^a^	4807
Maize	High	799	173^ab^	42	362	885	937^c^	678^a^	210^ab^	4086
	Low	884	128^a^	95	262	953	646^a^	629^a^	220^ab^	3818
Amylose	High	635	153^ab^	22	284	664	607^a^	626^a^	195^ab^	3186
	Low	1073	186^b^	64	280	845	739^ab^	704^a^	260^ab^	4150
SEM		51.23	6.93	7.76	12.36	44.17	32.13	38.26	26.18	380.5
Main effects: Starch										
Waxy rice		1022	174	45	332	1030^c^	873	789	224	4490^b^
Maize		841	151	69	312	919^b^	791	654	215	3952^ab^
Amylose		854	170	43	282	755^a^	673	665	227	3668^a^
Protein										
High		772^a^	172	32^a^	342^b^	837	807	631	223	3815
Low		1040^b^	157	73^b^	276^a^	966	751	774	222	4259
Significance (*P-*value)										
Starch		0.208	0.273	0.289	0.155	0.032	0.008	0.169	0.887	0.013
Protein		0.006	0.256	0.008	0.003	0.122	0.235	0.034	0.986	0.045
Starch × Protein		0.275	0.043	0.816	0.113	0.849	0.002	0.022	0.037	0.060

^d^Values are the mean and pooled standard error of the mean (SEM), *n* = 6 chicken/group

^a-c^means within each group with different letter designations differ (*P* < 0.05)

## Discussion

### Growth performance

Both slowly (amylose) and rapidly (waxy rice) digestible starch sources supported superior FCR than diets containing maize in this study. Starch digestion rates differ across feedstuffs [3, 12] and some studies have indicated that slowly digestible starch may advantage boiler growth performance [13–15]. Indeed, a high 90.4% of starch digestion along the small intestine took place in proximal jejunum (not shown) indicating that rapidly digestible starch enhanced FCR is ostensibly a curious outcome. However, Sydenham et al. [16] demonstrated that proximal jejunal starch/protein disappearance rate ratios in broilers quadratically influenced FCR and weight gain so it may not be prudent to consider starch digestion rates in isolation.

### mRNA expression for glucose transporters

The majority of glucose is co-absorbed with sodium from the gut lumen via *SGLT-1* transporters; whereas, *GLUT-2* provides the basolateral exit of glucose from enterocytes into the portal circulation [17]. *SGLT-1* is located in the apical membrane of enterocytes and *SGLT-1* mRNA expression occurs in response to high dietary carbohydrate levels [18]. In the present study, waxy rice in low protein diets generated the highest jejunal *SGLT-1* expression, which is not surprising as it is a rapidly digested starch source. However, this was not the case in high protein diets as there was a highly significant treatment interaction. That ileal *SGLT-1* expression was higher with waxy rice and maize diets than amylose may have been a compensatory mechanism due to the relative lack of starch and glucose in the ileum in comparison to amylose. *GLUT-2*, which is not a sodium dependent transporter, is located in the baso-lateral membrane of enterocytes and starch source was very influential in respect of mRNA expression. However, the mRNA response patterns in the two intestinal segments were almost exact opposites; waxy rice was highest in the jejunum and amylose was highest in the ileum. This difference may reflect the relative amounts of starch and glucose along the small intestine dependent of starch source and their digestion rates. As considered later, *GLUT-2* may be recruited into the apical membrane of enterocytes to amplify glucose absorption from the gut in certain physiological contexts [19].

### mRNA expressions for PepT-1 and amino acid transporters

Several proton-coupled oligopeptide transporters have been identified in poultry including peptide transporters 1 and 2 (*PepT1* and *PepT2*) and the peptide/histidine transporter 1 (*PHT1*) as reported by Zwarycz and Wong [20]. Relatively large quantities of amino acids as di- and tri-peptides can be absorbed rapidly via PepT1 and Krehbiel and Matthews [21] argued that the majority of amino acids is absorbed into enterocytes via *PepT1*. Therefore, it is noteworthy that *PepT1* mRNA expressions in the ileum (*r* = − 0.323; *P* = 0.077) tended to be associated with improvements in FCR of birds from 6 to 35 d post-hatch.

Relative mRNA expression for amino acid and oligopeptide transporters is a complex subject and dietary protein level, protein quality, feed restriction and bird age have all been shown to influence mRNA expression to variable extents [22–24]. In the present study, waxy rice starch generated the highest mRNA expressions. In contrast, the low dietary protein level increased ileal mRNA expressions of only two transporters, *EAAT3* and *LAT1*. According to Gilbert et al. [23], *B*^0^*AT1* is broad scope, Na^+^-dependent transporters of neutral amino acids. The *y*^*+*^*LAT1* transporter mediates Na^+^-dependent neutral amino acids and Na^+^-independent cationic amino acids; whereas, LAT1 mediates the Na^+^-independent transport of branched-chain and aromatic amino acids. Moss et al. [25] proposed that glucose and amino acids compete for intestinal uptakes via their respective Na^+^-dependent transporters. If so, this competition would take place mainly in the anterior small intestine with a rapid starch source such as waxy rice which may have resulted in a relative surplus of amino acids becoming available for absorption in the ileum; this may be reflected in the higher mRNA expressions for *ATB*^0,+^, *EAAT3*, *B*^0^*AT1* and *y*^*+*^*LAT1* with diets containing waxy rice. In the case of *B*^0^*AT1* and *y*^*+*^*LAT1*, waxy rice generated significantly higher mRNA expressions than maize or amylose. That dietary starch can influence mRNA expressions for amino acid transporters is intriguing and emphasises the interactions that may occur between starch and glucose with protein and amino acids.

### ATP, Na^+^/K^+^-ATPase and citrate synthase activities

The ‘sodium pump’, or Na^+^/K^+^-ATPase, is located in the baso-lateral membrane of enterocytes and drives the co-absorption of sodium with glucose or amino acids via Na^+^-dependent transporters. Instructively, diets containing amylose as a starch source had the highest sodium pump activities in the ileum. This suggests that amylose, a slowly digested starch, generated additional glucose to be absorbed in the ileum via *SGLT-1*. Interestingly, ileal sodium pump activity was positively correlated (*r* = 0.406; *P* < 0.025) with weight gains and tended to be negatively correlated (*r* = − 0.324; *P* = 0.062) with FCR from 21 to 35 d post-hatch. That sodium pump activity in the ileum was associated with enhanced broiler performance illustrates the importance of intestinal uptakes of nutrients for the performance of broiler chickens [26]. Starch source and protein level had tangible, significant impacts on citrate synthase activity in both small intestinal segments; in contrast; they did not influence ATP contents. This is surprising given that citrate synthase is a pivotal enzyme in the tricarboxylic acid cycle which generates ATP [27]. When glucose is a limiting factor, many anaplerotic amino acids can supply OAA and acetyl-CoA, providing support for the TCA cycle. The higher activity levels of CS in the intestinal mucosa of amylose-fed animals suggests that nutrient-limited conditions may require cells to engage alternative metabolic pathways, including degradation of proteins, to supply carbon to the TCA cycle [28].

### Proliferation, apoptosis and mRNA expression for *CASP-3* and *mTOR*

Dietary carbohydrate sources might influence rates of cell proliferation and apoptosis or programmed cell death in the gut mucosa and the measurement of BrdU positive cells is a means to detect proliferating cells along the small intestine. Diets containing waxy rice as a starch source generated the highest numbers of BrdU positive cells in the jejunum, which suggests that a rapidly digestible starch source promotes development of the anterior small intestine. Somewhat reciprocally, waxy rice diets generated the highest mRNA expression for *CASP-3* in the ileum, which is indicative of apoptosis. Apoptosis-induced proliferation maintains tissue homeostasis as dying cells induce proliferation of the surviving cells to compensate for the tissue loss and restore organ size [29]. Also, suppressed ileal mRNA expression of *CASP-3* by amylose suggests less apoptosis of enterocytes in the ileum which is consistent with an increased load of slowly digestible starch reaching the posterior small intestine. Diets containing amylose as a starch source generated the highest mRNA expression for *mTOR* in the ileum which was more pronounced in low protein diets. One interpretation of this outcome is that a slowly digestible starch source was associated with increased protein synthesis and cell proliferation in the ileal gut mucosa [30–32].

### Intestinal GOT activities and free amino acid concentrations in portal circulation

Glutamic acid is almost certainly the most catabolised amino acid in the gut mucosa for energy provision and GOT activity is indicative of the extent of that catabolism [33]. Ileal GOT activity tended to decline (*P* = 0.057) when either maize or amylose were the starch sources in comparison to waxy rice which is consistent with the proposition that rapidly digestible provides little glucose as an energy substrate to the posterior small intestine resulting in more catabolism of amino acids, especially glutamic acid. The significant increase in GOT with the transition from high to low protein diets indicates greater catabolism of glutamic acid which may have been a compensatory response to mitigate the catabolism of essential amino acids.

The pivotal question, whether or not amino acid catabolism in the gut mucosa is subject to nutritional regulation, was posed by Reeds et al. [9]. The outcomes of this study indicate that free amino acid concentrations in the portal circulation can be modified by dietary strategies. As a main effect, starch source influenced glutamine concentrations where waxy rice supported higher levels than maize and amylose. This was the only significant impact of starch source observed and may related to the fact that glutamine entry into enterocytes from the arterial circulation exceeds that of other amino acids [34]. As main effects, protein levels significantly influenced concentrations of six amino acids and significant treatment interactions were observed for 10 amino acids from the total of 18 assessed. Thus dietary treatments, significantly influenced the concentrations of free amino acids in plasma taken from the anterior mesenteric vein for 17 of the 18 amino acids assessed, the exception was tryptophan. However, the outcomes are inconsistent and interpretations are not straightforward.

Nevertheless, Li et al. [35] concluded that dietary sources of starch profoundly affect the net appearance of amino acids and glucose in the portal vein of growing pigs and that starch source has important implications for the efficiency of nutrient utilisation. Concentrations of non-essential amino acids in the portal circulation are more dominant. Therefore, it is noteworthy that waxy rice as a starch source generated 13.6% numerically higher concentrations than maize and 22.4% significantly higher concentrations of non-essential amino acids than amylose in this study. This outcome conflicts with the findings of van der Meulen et al. [36] who reported that slowly digestible starch (peas) increased the net portal flux of amino acids more than relatively rapidly digestible starch (maize) in pigs. However, it is in agreement with the report by Yin et al. [37] who found that rapidly digestible starch increased post-prandial concentrations of free amino acid in the systemic circulation of pigs. These researchers conceded that the precise mechanisms responsible have yet to be clarified but suggested that “rapidly digestible starch ameliorates the digestive and absorptive function” which promotes higher systemic circulating concentrations of most amino acids within four hours post-prandially.

As mentioned earlier, *GLUT-2* may be recruited into the apical membrane of enterocytes to amplify glucose absorption from the gut [19]. There is the possibility that rapidly digestible waxy rice starch triggered this recruitment in the anterior small intestine to absorb glucose which would reduce the quantity of glucose absorbed via the Na^+^-dependent transporter *SGLT-1*. This in turn would allow greater co-absorption of amino acids and sodium and ameliorate any competition for intestinal uptakes between glucose and amino acids. Thus, the apical recruitment of *GLUT-2* may be the genesis of the responses observed by Yin et al. [37] in pigs and the present study in poultry. While speculative, it may have been that neither pea nor maize starch prompted the apical recruitment of *GLUT-2* in the van der Meulen et al. [36] study resulting in the dichotomy of outcomes.

## Conclusions

Efficient FCR is vital for sustainable chicken-meat production and that both rapidly and slowly digestible starch enhanced FCR is an intriguing finding. In response to question posed by Reeds et al. [9], the outcomes of this study indicate that amino acid catabolism in the gut mucosa is subject to nutritional regulation. Given that amino acids can be spared from catabolism in the gut mucosa by supplementation of amylose, it follows their post-enteral availability would be improved and intestinal energy would be derived more efficiently from glucose.

## Abbreviations

AA: Amino acid
APN: Aminopeptidase
ATB^0,+^: Solute carrier family 6 member 14
ATP: Adenosine triphosphate
B^0^AT1: Solute carrier family 6 member 19
CASP-3: Caspase-3
CAT1: Solute carrier family 7 member 1
CS: Citrate synthase
EAAT3: Solute carrier family 1 member 1
FCR: Feed conversion ratio
GLUT-2: Glucose transporter 2
GOT: Glutamic-oxaloacetic transaminase
LAT1: Sodium-independent neutral amino acid transporter
mTOR: Mammalian target of rapamycin
OAA: Oxaloacetic acid
PepT-1: Peptide transporter 1
pGI: Potential glycaemic index
rBAT: Solute carrier family 3 member 1
SGLT-1: Sodium-glucose linked transporter
TCA: Tricarboxylic acid
y^*+*^LAT1: Solute carrier family 7 member 7
